# Pharmacokinetics of cannabidiol-/cannabidiolic acid-rich hemp oil in juvenile cynomolgus macaques (*Macaca fascicularis*)

**DOI:** 10.3389/fvets.2023.1286158

**Published:** 2023-11-29

**Authors:** Tinika N. Johns, Joseph J. Wakshlag, Alexander V. Lyubimov, Alexander Zakharov, Wesley M. Burnside

**Affiliations:** ^1^Haman Ranch, The Mannheimer Foundation, LaBelle, FL, United States; ^2^Department of Clinical Sciences, College of Veterinary Medicine, Cornell University, Ithaca, NY, United States; ^3^Toxicology Research Laboratory, Department of Pharmacology, College of Medicine, University of Illinois at Chicago, Chicago, IL, United States

**Keywords:** pharmacokinetics, monkey, nonhuman primate, cannabidiol, cannabidiolic acid, cannabinoids, hemp (*Cannabis sativa* L.), noncompartmental analysis

## Abstract

**Introduction:**

Cannabinoids are increasingly popular in human and veterinary medicine and have been studied as an alternative treatment for a wide range of disorders. The goal of this study was to perform a pharmacokinetic analysis of oral cannabidiol (CBD)-/cannabidiolic acid (CBDA)-rich hemp oil (CBD/ArHO) in juvenile cynomolgus macaques (*Macaca fascicularis*).

**Methods:**

After a 2 mg/kg CBD/ArHO pilot study, 4 and 8 mg/kg direct-to-mouth CBD/ArHO were administered (*n* = 4 per dose) once daily for 14 days and blood was collected at 0-, 0.5-, 1-, 2-, 4-, 8-, 12-, and 24-h, and on Days 7 and 14, to quantify serum cannabinoid concentrations by high-performance liquid chromatography–tandem mass spectrometry. Serum biochemistries and complete blood counts were performed on Days 0, 1, and 14.

**Results:**

The maximum mean serum concentration (C_max_) of CBDA was 28.6–36.2 times that of CBD at 4 and 8 mg/kg. At 8 mg/kg, the C_max_ of CBD was 1.4 times higher (*p* = 0.0721), and CBDA was significantly 1.8 times higher (*p* = 0.0361), than at 4 mg/kg. The maximum mean serum concentration of ∆^9^-tetrahydrocannabinol (THC) was 4.80 ng/mL at 8 mg/kg. Changes in serum biochemistries and complete blood counts over time were not clinically significant.

**Discussion:**

Given the low serum CBD concentrations, the doses and frequency used in this study may be insufficient for a therapeutic effect of CBD in particular; therefore, clinical studies are needed to determine the therapeutic dose of CBD and CBDA for macaques, which may differ based on the disorder targeted.

## 1 Introduction

The hemp plant (*Cannabis sativa L*.) is a source of a variety of cannabinoids, with the most notable abundant bioactive components being cannabidiol (CBD), ∆^9^-tetrahydrocannabiol (THC), and their acids (1). Temperature, humidity, precipitation, and genetic variety affect the phytocannabinoid profile of hemp plants (2), wherein cannabigerolic acid (CBGA) is converted to cannabidiolic acid (CBDA) by CBDA synthase or to ∆^9^-tetrahydrocannabinolic acid (THCA) by THCA synthase (3, 4). Then, light, heat, or oxygenation cause decarboxylation of these acids to cannabigerol (CBG), CBD, or THC during storage and processing (5, 6). Although CBD can be converted to THC under artificial gastric conditions *in vitro* (7), studies in humans and other species have failed to demonstrate this conversion when CBD is administered orally (8–10). The hemp plant also produces other phytochemicals, such as terpenes and hydrocarbons, that may potentiate cannabinoid activity—known as the *entourage effect* (11).

Due to its psychoactive properties, THC is currently a schedule I controlled drug in the United States except for a few synthetic prescription-only forms (12, 13); however, there is a lack of federal regulation regarding the sale and use of CBD-containing products. The Agricultural Improvement Act of 2018 resulted in federal legalization of cannabinoid products containing less than 0.3% THC (14). In addition, other commercially available products widely vary in CBD concentration, recommended dose, and routes of administration. Independent analyzes of numerous products have also demonstrated inconsistencies between the CBD concentrations provided on the label and their actual contents (15, 16).

While the only current FDA-approved use for CBD is as an anticonvulsant to treat seizures associated with Lennox-Gastaut syndrome, Dravet syndrome, or tuberous sclerosis complex (17), recent studies have indicated additional therapeutic benefits of CBD including anti-inflammatory (18), antioxidant (19, 20), analgesic (21), antiemetic (22), anxiolytic (23), anticarcinogenic (24, 25), antimicrobial (26, 27), and immunomodulatory (21, 28) properties. More specifically, research in various species has evaluated its efficacy in treating diseases such as diabetes mellitus (29–31), osteoarthritis (21, 28, 32), and neurologic diseases (e.g., Alzheimer’s, Parkinson’s) (33–37). In addition, CBD has been proposed to treat symptoms associated with stress (38), gastrointestinal disturbances (39, 40), orofacial pain (41, 42), hypertension (43–45), and neoplastic processes (46). Prior to cisplatin chemotherapy in the house musk shrew (*Suncus murinus*; *n* = 20), intraperitoneal CBD demonstrated a biphasic response—low doses (5 mg/kg) reduced emesis and higher doses (40 mg/kg) potentiated it (22); similarly, prior to lithium chloride administration (*n* = 36), intraperitoneal CBDA significantly reduced the incidence of emesis at low doses (0.1–0.5 mg/kg) but increased dosing (5 mg/kg) did not significantly reduce emesis compared to the control (47). Far fewer studies have been conducted regarding the other therapeutic benefits of CBDA but include anti-inflammatory (48), analgesic (49, 50), anticonvulsant (51), and anxiolytic (52–54) properties. Compared to CBD, CBDA was a more potent antihyperalgesic (49) but was less effective in reducing cancer cell proliferation *in vitro* (24). Unheated hemp extract contained higher concentrations of CBDA than CBD but resulted in a higher maximum serum CBD concentration compared to heated hemp extract (1).

Overall, CBD is reportedly safe with minimal side effects or signs of toxicity. Most negative effects have been anecdotal, inconsistent among studies, or occurred with high doses or extended use. Rarely reported potential side effects include sedation, mild diarrhea, inappetence, agitation, hypersensitivity, poor sleep quality, ataxia, pyrexia, infection, dry mouth, head-shaking, and excessive licking (55–59). Mildly elevated serum liver enzymes, including alanine transaminase, alkaline phosphatase (ALP), and aspartate aminotransferase (AST), were reported at typical daily CBD doses (0.5–2.8 mg/kg) in some human, canine, and feline subjects (28, 60, 61); although the increases were statistically significant, they were not of clinical concern. Conversely, higher CBD doses (≥ 20 mg/kg) in humans may lead to drug-induced liver injury (62, 63). There is also evidence that CBD may interfere with some drug metabolism by inhibition of cytochrome P450 enzymes (64, 65). Therefore, caution should be used prior to concurrent administration with other drugs, especially those with hepatic metabolism or affected by cytochrome P450 inhibition.

There are no current studies published about the use of CBD or CBDA as a potential therapeutic agent in nonhuman primates (NHP); however, oral high-dose CBD (30–300 mg/kg) for 90 days (*n* = 16) did not affect rhesus macaque growth rates but increased liver and kidney weights, decreased testicular weight and inhibited spermatogenesis, dose-dependently decreased red blood cell counts, and occasionally resulted in transient diarrhea (66). Based on evidence of the therapeutic benefits in humans and other species (32, 38, 40), low-dose CBD could be a candidate as an adjunctive therapy for some common NHP medical conditions, including osteoarthritis (67), environmental stress (68), and inflammatory diarrheal disease (69). As a first step, a pharmacokinetic (PK) analysis of CBD and CBDA would inform whether doses recently reported in other species would be appropriate in macaques and provide a baseline for future studies evaluating its PK or therapeutic potential.

Based on the therapeutic doses of CBD and CBDA reported in other species (28, 55, 61, 70, 71), we selected a commercially available oral veterinary CBD-/CBDA-rich hemp oil (CBD/ArHO; approximately 1:1 CBD:CBDA) with a guaranteed content analysis (Supplementary Table S1) that was previously evaluated in domestic dogs (70). We also performed a pilot study at 2 mg/kg CBD/ArHO in cynomolgus macaques and determined that serum CBD concentrations were lower than in some species, but serum CBDA concentrations were 3.8 times higher than CBD (72). The goal of this study was to perform a PK analysis of CBD/ArHO in juvenile cynomolgus macaques (*Macaca fascicularis*). Our objectives were to (1) perform a 24-h, single-dose PK study at 4 or 8 mg/kg CBD/ArHO; (2) determine any physical, biochemical, or hematological effects over time; (3) determine if any cannabinoids accumulate in the serum after 14 days of daily dosing, and (4) compare the results to reported findings in other species. We hypothesized that 8 mg/kg CBD/ArHO would provide higher serum concentrations of CBD and CBDA than 4 mg/kg, with negligible amounts of serum THC, and CBDA being significantly higher than CBD. We also hypothesized that there would be minimal negative physical effects, and no clinically significant serum biochemical or hematological changes.

## 2 Materials and methods

### 2.1 Humane animal care and use

All procedures were approved by The Mannheimer Foundation IACUC, an AAALAC-accredited facility, and adhered to all approved standard operating procedures. Animals were maintained according to Animal Welfare Act (73, 74) and Regulations (75), and the *Guide for the Care and Use of Laboratory Animals* (76).

### 2.2 Animals

Male [*n* = 4; weight (mean ± 1 SD), 3.70 ± 0.67 kg] and female (*n* = 4; weight, 3.63 ± 0.32 kg) juvenile cynomolgus macaques (aged 3.29 ± 0.13 years) were selected. Inclusion criteria were subjects born at The Mannheimer Foundation, open to positive human interactions (determined by colony manager), and naïve to experimental and medical interventions outside of the routine preventative medicine program. This program included semiannual physical examination, tuberculin skin testing (10 μL intradermally every 6 months; Tuberculin OT, Colorado Serum Company, Denver, CO), deworming with ivermectin [0.4 mg/kg intramuscularly (IM) every 6 months; Vetrimec 1%, MWI Animal Health, Boise, ID], and routine vaccination against *Clostridium tetani* (0.5 mL IM every 5 years; tetanus toxoid, Fort Dodge Animal Health, Fort Dodge, IA), *Measles morbillivirus* [1 mL subcutaneously (SC) every 6 months; Vanguard DM, Zoetis, Parsippany, NJ], and *Rabies lyssavirus* (1 mL SC every 3 years; Rabvac 3, Elanco US, Fort Dodge, IA). Animals were serologically negative for *Macacine herpesvirus 1*, *Simian retrovirus 1*, *Simian T-lymphotrophic virus 1*, and *Simian immunodeficiency virus*, and identified by a SC passive integrated transponder (PetLink™ Slim, Datamars, Inc., Woburn, MA) from birth and a chest tattoo of their unique identification number after 6 months old.

Subjects were individually caught in nets, anesthetized with ketamine hydrochloride (10–15 mg/kg IM), boxed (Prima-Carrier, Primate Products, Immokalee, FL), and transferred from outdoor, same-sex and -age social housing units. Animals were weighed, physically examined, collared (medium, aluminum; Primate Products, Immokalee, FL), and same-sex pair-housed in 2 stainless-steel squeeze-back cages with an open pass-through door (floor area, 0.4 m^2^ each; height, 76.2 cm) in indoor, climate-controlled rooms [70.3–80.2°F (21.3–26.8°C); relative humidity, 40–95%] on a 12:12-h light:dark cycle (07:00–19:00); cages were sanitized daily and disinfected at least every 15 days. Animals were fed a standard commercial primate diet (5049, Lab Diet, St Louis, MO) twice daily, and watered free-choice through an automated watering system. Each animal was environmentally enriched with a mirror, plastic ball, foraging board, and daily forage, including seeds, fruit, popcorn, multigrain fruit cereal (Fruit Spins, Great Value, Bentonville, Arkansas), and FiberBites (ClearH2O, Westbrook, ME). Animals were observed at least once daily to assess and document mentation, feed consumption, hydration status, stool quality, and any behavioral or health concerns.

Subjects were individually acclimated to human interactions and trained by positive reinforcement with handfed forage at least once daily after temporary separation by closing the mesh pass-through door. Soft classical music was played during handling. After the animals readily took forage directly from the trainers’ hand, they were acclimated to the pole (Primate Products, Immokalee, FL). Once the animal ignored the pole when held by the trainers, the pole was latched transversely through the cage mesh until it was accepted as a neutral object. Then, the pole was repeatedly held as close to the collar as the animal would allow until it could be latched to the collar. Once latching and unlatching of the pole to the collar was repeatedly successful, the animals were trained to allow two trainers to each latch their pole on opposite sides of the collar, climb from their cage to the floor, sit on the primate restraint chair (Primate Products, Immokalee, FL) in the same animal room, and allow their collar to be secured into the chair for 10–15 min intervals. After study completion, macaques were anesthetized again with ketamine hydrochloride for collar removal, weighed, boxed, and returned to their outdoor enclosures.

### 2.3 Study design

**Figure 1:**
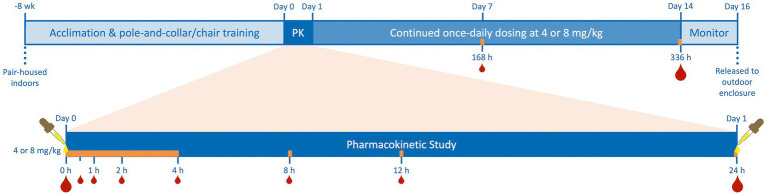
Timeline of juvenile cynomolgus macaque (4 males, 4 females) pair-housing, indoor acclimation, and pole-and-collar/chair training, 24-h pharmacokinetic study (PK) after single-dose oral administration of cannabidiol-/cannabidiolic acid-rich hemp oil (Day 0) at 4 or 8 mg/kg (2 males and 2 females per dose), continued once-daily dosing for an additional 13 days, and a two-day period of monitoring prior to release to their outdoor enclosures. Subjects were dosed daily at the same time as on their individual Day 0 and blood was always drawn immediately following the dose on Days 1, 7, and 14 as on Day 0. Blood was collected at 0-, 0.5-, 1-, 2-, 4-, 8-, 12-, 24-, 168-, and 336-h to quantify serum cannabinoid concentrations by high-performance liquid chromatography–tandem mass spectrometry. Additional blood was drawn for serum biochemistries and complete blood counts on Days 0, 1, and 14 (indicated by the larger blood drops). The orange bar represents the times of chair restraint for blood collection during the study, never exceeding 4 h.

Eight subjects were selected to receive 4 (2 male, 2 female) or 8 (2 male, 2 female) mg/kg of CBD/ArHO (70 mg/mL). Then, a randomized block design was applied. Animals were grouped by sex before being randomly assigned to dose groups using a random sequence generator;^1^ the order of subject dosing was also randomized. The CBD/ArHO was administered orally with a 12-gauge curved gavage needle and 1-mL syringe after chairing. Subsequently, each animal was accessed by employing the squeeze-back mechanism on cage to allow for daily dosing for the study duration unless the animal was chaired for blood collection. All doses of CBD/ArHO (ElleVet Sciences, South Portland, ME) were administered between 07:00 and 09:00 on a given day prior to feeding. After a maximum chairing time of 4 h, animals were returned to their individual enclosures until the next timepoint for sample collection. Feed was provided immediately after the first 4 h of chairing. CBD/ArHO was administered for 14 days to determine any acute or subacute physiologic parameters over time, or if there was a cumulative effect of repeated administration. A procedural timeline is provided in Figure 1.

Immediately following CBD/ArHO administration, blood was collected at Days 0 (0 h), 1 (24 h), and 14 (336 h) by femoral venipuncture using a 22-gauge vacuum phlebotomy system (Vacutainer, BD, Franklin Lakes, NJ), 4-mL serum-separator tube (8881302072, Medtronic, Minneapolis, MN), and 3-mL K2-EDTA tube (367856, Vacutainer, BD, Franklin Lakes, NJ). The serum tubes were held at 4°C for at least 30 min before centrifugation at 1,300 × *g* for 15 min at 4°C. The serum was divided into two 2-mL screw-top microtubes (72.694.406, Sarstedt, Nümbrecht, HR); one was stored at −80°C for PK analysis, while the other was refrigerated at 4°C with the K2-EDTA tube for serum biochemistry and complete blood count, respectively. Additional blood was collected at 0.5, 1, 2, 4, 8, and 12 h, and Day 7 (168 h) from alternating saphenous veins with a 25-gauge needle and 1-mL luer-lock syringe (ML12558, Air-Tite Products, Virginia Beach, VA); a cephalic or femoral vein was used if the saphenous collection failed. The blood was transferred to a 0.5-mL serum separator tube (365967, Becton Dickinson, Franklin Lakes, NJ) and held at 4°C for at least 30 min before centrifugation at 1,300 × *g* for 15 min at 4°C. Serum samples were transferred into sterile 2-mL screw-top microtubes and stored at −80°C until all samples were submitted for PK analysis.

### 2.4 Sample analysis

All samples were wrapped in an absorbable pad, placed in a 1-gallon Ziploc Freezer bag, and packaged in an insulating extruded polystyrene box within a cardboard box for overnight shipment according to the requirements of the International Air Transport Association Dangerous Goods Regulations for Category B biologic substances (UN 3373). Refrigerated serum and K2-EDTA samples were shipped with cold packs to VRL Laboratories (San Antonio, TX) for biochemistry and complete blood count (5506, Chem Profile II and CBC) within 48 h. Serum samples for cannabinoid analysis were shipped in dry ice (UN 1845) to the University of Illinois at Chicago Toxicology Research Laboratory.

Serum cannabinoid analyzes were performed using an exploratory (fit-for-purpose) method for fast measurement of 11 cannabinoids and their metabolites. The reference standards for CBD and CBDA were obtained from Restek Corporation (Bellefonte, PA); all other reference and internal standards were obtained from Cerilliant Corporation (Round Rock, TX). Cannabinoid serum concentration for CBD, CBDA, THC, THCA, cannabinol (CBN), cannabichromene, CBG, and CBGA and their metabolites 7-Nor-7-carboxycannabidiol (7-COOH-CBD), 11-nor-9-Carboxy-Δ9-tetrahydrocannabinol (COOH-THC), and 11-nor-9-Carboxy-Δ9-tetrahydrocannabinol-Glucuronide (COOH-THC-Glu) was determined using high-performance liquid chromatography–tandem mass spectrometry (Nexera X2 and LCMS 8050, Shimadzu Corp., Kyoto, Japan).

Each serum sample (40 μL) was mixed with internal standards (20 μL, 100 ng/mL each) in 1:1 water:methanol in a 96 well plate. Then, proteins were precipitated, and compounds were extracted by adding ice-cold acetonitrile (80 μL), vortexing for 1–2 min, and centrifuging at 4,000 rpm for 10 min at 4°C. Supernatants (70 μL) were diluted with deionized water (70 μL) in a another 96 well plate and centrifuged again. 10 μL of the processed samples were injected into a column (100 Å, 3 μm, 2.1 × 50 mm, Atlantis T3 column, Waters, Milford, MA) coupled to liquid chromatography–tandem mass spectrometry. A guard cartridge (100 Å, 3 μm, 2.1 × 5 mm, Atlantis T3 VanGuard, Waters, Milford, MA) was also used for columnar protection. The column was equilibrated with mobile phase A (0.1% formic acid in water) and mobile phase B (acetonitrile) at 50% B. The compounds were eluted by a linear gradient from 50% B to 95% B over 6 min, and then held at 95% B for 1 min. Subsequently, the column was reequilibrated at initial composition for 1 min at a flow rate of 0.3 mL/min. The autosampler and column temperature were set a 4°C and 30°C, respectively. The cannabinoids and their metabolites were detected in electrospray ionization positive or negative mode. Interface voltage was 4 kV or − 3.5 kV. Interface, desolvation line, and heat block temperatures were 300, 250, and 400°C, respectively. Nebulizing, heating, and drying gas flow were 2.7, 5, and 5 L/min, respectively. Serum cannabinoids concentrations were calculated by LabSolutions software (Shimadzu Corp., Kyoto, JP) using a quadratic calibration curve with 1/concentration^2^ weighing based on relative response (peak area of cannabinoids/peak area of internal standards). The reference standards, their multiple reaction monitoring, polarity, and retention time, internal standards and their multiple reaction monitoring and polarity, and calibration curve range are summarized in Table 1.

**Table 1:** High-performance liquid chromatography–tandem mass spectrometry reference standards (RS) for cannabidiol (CBD), cannabidiolic acid (CBDA), (−)-∆^9^-tetrahydrocannabinol (THC), ∆^9^-tetrahydrocannabinolic acid A (THCA), cannabigerol (CBG), cannabigerolic acid (CBGA), 7-Nor-7-carboxycannabidiol, (7-COOH-CBD), (+)-11-nor-9-Carboxy-Δ^9^-THC (COOH-THC), (+)-11-nor-9-Carboxy-Δ^9^-THC-Glucuronide (COOH-THC-Glu), cannabichromene (CBC), and cannabinol (CBN) with multiple reaction monitoring (MRM) and polarity, and retention time, as well as internal standards with MRM and polarity, and the overall calibration curve range (lower and upper limits of quantification) are presented.

Reference standard				Internal standard		Calibration curve range (ng/mL)
Cannabinoid	Catalog number	MRM (Polarity)	Retention time	Name	MRM (Polarity)	
CBD	34,011	315 > 193 (+)	4.55	CBD-d3	318 > 196 (+)	2.5–1,000
CBDA	34,099	359 > 219 (+)	4.20	CBD-d3	318 > 196 (+)	1–2,500
THC	T-005	315 > 193 (+)	5.60	THC-d3	318 > 196 (+)	1–1,000
THCA	T-093	357 > 245 (−)	6.10	THCA-d3	357 > 248 (−)	1–1,000
CBG	C-141	317 > 193 (+)	4.45	CBD-d3	318 > 196 (+)	1–1,000
CBGA	C-142	361 > 219 (+)	4.35	CBD-d3	318 > 196 (+)	1–1,000
7-COOH-CBD	B140796	343 > 299 (−)	2.25	7-COOH-CBD-d3	346 > 302 (−)	1–1,000
COOH-THC	T-006	345 > 299 (+)	3.55	COOH-THC-d9	354 > 308 (+)	1–250
COOH-THC-Glu	T-038	519 > 345 (−) 521 > 345 (+)	2.00–2.10 (RS) 2.15–2.25 (Animals)	COOH-THC-Glu-d3	522 > 346 (−) 524 > 348 (+)	1–250
CBC	C143	315 > 193 (+)	5.95	THC-d3	318 > 196 (+)	2.5–1,000
CBN	C-046	311 > 223 (+)	5.20	CBD-d3	318 > 196 (+)	1–1,000

### 2.5 Pharmacokinetic analysis

The PK analyzes were performed using Phoenix WinNonlin™ v8.3 (Certara, Princeton, NJ) by employing a plasma model (200–202) with extravascular dosing and the best fit method, which calculated the coefficient of determination (R^2^), maximum serum concentration (C_max_; ng/mL), time to maximal serum concentration (T_max_; h), and half-life of the terminal phase (*t*_1/2–λz_; h); a linear trapezoidal linear interpolation was used to calculate the area under the curve until the last measurement (AUC_last_; h·ng/mL), area under the moment curve until the last measurement (AUMC_last_; h^2^·ng/mL) and mean residence time until the last measurement (MRT_last_; h). All values below the quantification level (BQL) before C_max_ were set to zero. The first value BQL after C_max_ was calculated as half of the lower limit of quantification (Table 1) for each cannabinoid; the subsequent values BQL were set to zero. Based on a limited sample size, lack of statistical difference by sex, and relatively low serum cannabinoid concentrations resulting in too few data points to calculate all non-compartmental analysis parameters by individual, the mean data were used for group analysis.

### 2.6 Statistical analysis

Mixed-effects models with the Geisser–Greenhouse correction were performed using Prism v10.0.0 for macOS X (GraphPad, Boston, MA) to determine statistical significance (*p* ≤ 0.05). Random effects zero or less were removed to simplify the model. The independent variables included CBD/ArHO dose (mg/mL), sex (male, female), and time (h). Dependent variables included serum cannabinoid concentrations (ng/mL), serum biochemical concentrations, and complete blood count parameters, which were square-root transformed to improve normality. Residual plots were used to confirm model correctness and Quantile-Quantile plots of predicted versus actual residuals were used to confirm distribution normality.

## 3 Results

### 3.1 Pharmacokinetic study

All animals were bright, alert, and responsive throughout the study. During daily observations, they consistently maintained normal hydration, appetites, and stool quality. Most animals were reluctant to take the CBD/ArHO, many of whom turned their heads away from the gavage needle and syringe, and actively pushed them away during administration. Immediately following administration of the CBD/ArHO, some animals had mild hypersalivation, which resolved prior to the next blood collection timepoint.

#### 3.1.1 Serum cannabinoids

**Figure 2:**
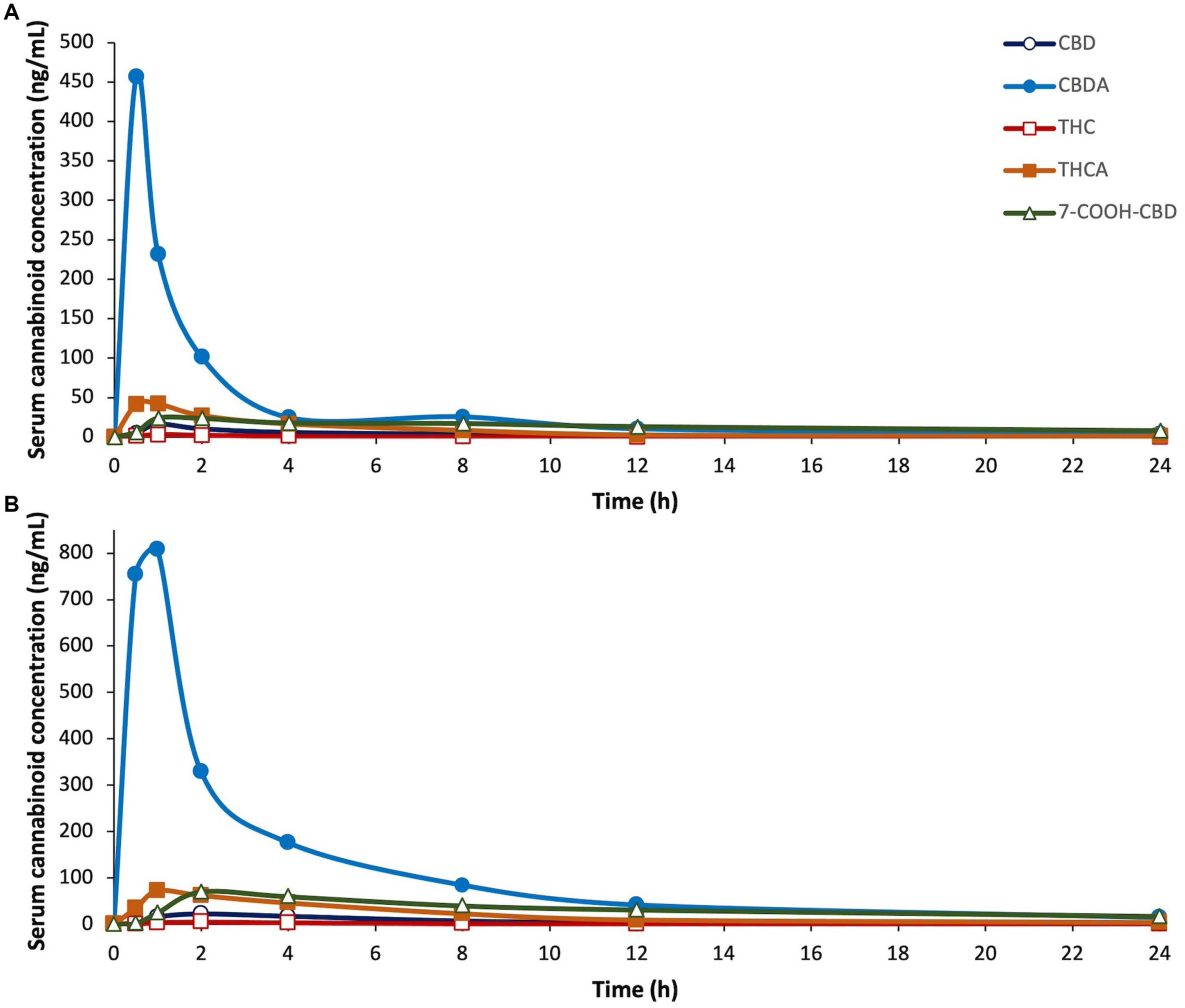
Serum concentrations of cannabidiol (CBD), cannabidiolic acid (CBDA), ∆^9^-tetrahydrocannabinol (THC), tetrahydrocannabinolic acid (THCA), and 7-carboxy cannabidiol, (7-COOH-CBD), after a single dose of oral administration of cannabidiol-/cannabidiolic acid-rich hemp oil at **(A)** 4 or **(B)** 8 mg/kg to juvenile cynomolgus macaques (*n* = 4 per dose) over 24 h. Note differences in the X-axes due to the sizeable increases in serum cannabinoid concentrations between doses. Each cannabinoid significantly differed over time (*p* ≤ 0.0017).

Each cannabinoid significantly differed over time (*p* ≤ 0.0017). There were no significant differences between males and females for any of the cannabinoids. CBDA was the only cannabinoid that significantly differed by dose over time (*p* = 0.0361); the 8 mg/kg dose was significantly higher than the 4 mg/kg dose. The PK curves for CBD, CBDA, THC, THCA, and 7-COOH-CBD are displayed in Figure 2. The PK results for all detectable cannabinoids are summarized in Table 2 and the serum cannabinoid concentrations on Days 1, 7, and 14 are summarized in Table 3.

**Table 2:** Pharmacokinetic summary of cannabidiol (CBD), cannabidiolic acid (CBDA), ∆^9^-tetrahydrocannabinol (THC), ∆^9^-tetrahydrocannabinolic acid (THCA), cannabigerolic acid (CBGA), 7-Nor-7-carboxycannabidiol (7-COOH-CBD), 11-nor-9-Carboxy-Δ^9^-tetrahydrocannabinol (COOH-THC), and 11-nor-9-Carboxy-Δ^9^-tetrahydrocannabinol-Glucuronide (COOH-THC-Glu) after a single dose of oral administration of cannabidiol-/cannabidiolic acid-rich hemp oil at 4 or 8 mg/kg to juvenile cynomolgus macaques (*n* = 4 per dose) over 24 h.

Cannabinoid	R^2^	C_max_ (ng/mL)	T_max_ (h)	*t*_1/2–λz_ (h)	AUC_last_ (h·ng/mL)	AUMC_last_ (h^2^·ng/mL)	MRT_last_ (h)
4 mg/kg							
CBD	0.9124	15.98 ± 11.07	1	5.57	75.12	437.71	5.83
CBDA	0.9417	456.75 ± 187.48	0.5	6.80	838.28	3264.70	3.89
THC	0.9989	3.94 ± 2.42	1	1.94	12.50	33.03	2.64
THCA	0.9464	41.90 ± 12.77	1	4.60	204.62	1002.26	4.90
CBGA	0.8446	12.14 ± 6.92	0.5	2.20	18.82	40.81	2.17
7-COOH-CBD	0.9909	24.19 ± 15.30	1	15.11	335.16	3304.96	9.86
COOH-THC	0.9727	3.74 ± 2.08	2	11.30	27.33	218.66	8.00
COOH-THC-Glu	0.5460	9.18 ± 4.95	2	12.76	19.15	663.43	8.66
8 mg/kg							
CBD	0.9735	22.31 ± 5.90	2	5.81	157.05	943.76	6.01
CBDA	0.9736	807.33 ± 281.65	1	6.54	2759.26	12634.97	4.58
THC	0.8495	4.80 ± 1.12	2	4.51	26.36	131.61	4.99
THCA	0.9427	71.90 ± 25.65	1	5.36	485.25	2916.39	6.01
CBGA	0.9543	18.23 ± 6.61	1	8.66	63.37	378.14	5.97
7-COOH-CBD	0.9979	70.31 ± 31.86	2	13.34	817.24	7698.93	9.42
COOH-THC	0.5539	8.02 ± 3.54	2	9.69	56.14	425.79	7.58
COOH-THC-Glu	0.8082	19.98 ± 9.03	4	20.34	21.16	1495.14	8.83

The goodness-of-fit (R^2^), mean maximum serum concentration (C_max_; mean ± SEM), time to maximum serum concentration (T_max_), half-life of the terminal phase (t_1/2–λz_), area under the curve until the last measurement (AUC_last_), area under the moment curve until the last measurement (AUMC_last_), and mean residence time (MRT_last_) are presented.

**Table 3:** Mean serum cannabinoid concentration (mean ± SEM) of cannabidiol (CBD), cannabidiolic acid (CBDA), ∆^9^-tetrahydrocannabinol (THC), ∆^9^-tetrahydrocannabinolic acid (THCA), cannabigerolic acid (CBGA), 7-Nor-7-carboxycannabidiol (7-COOH-CBD), 11-nor-9-Carboxy-Δ^9^-tetrahydrocannabinol (COOH-THC), and 11-nor-9-Carboxy-Δ^9^-tetrahydrocannabinol-Glucuronide (COOH-THC-Glu) after once daily oral administration of cannabidiol-/cannabidiolic acid-rich hemp oil at 4 or 8 mg/kg to juvenile cynomolgus macaques (*n* = 4 per dose) on Days 1, 7, and 14 at 24, 168, and 336 h, respectively.

Cannabinoid	Serum Concentration (ng/mL)					
	Day 1		Day 7		Day 14	
	4 mg/kg	8 mg/kg	4 mg/kg	8 mg/kg	4 mg/kg	8 mg/kg
CBD	BQL	BQL	BQL	3.40 ± 0.34	BQL	1.71 ± 1.71
CBDA	3.74 ± 0.63	14.25 ± 3.68	3.19 ± 1.63	18.15 ± 7.15	2.56 ± 0.73	8.50 ± 3.59
THC	BQL	BQL	BQL	BQL	BQL	BQL
THCA	0.98 ± 0.18	3.73 ± 0.89	0.37 ± 0.37	2.86 ± 0.57	0.64 ± 0.39	1.55 ± 0.58
CBGA	BQL	0.66 ± 0.26	BQL	0.28 ± 0.28	BQL	BQL
7-COOH-CBD	8.10 ± 2.76	17.20 ± 5.58	13.76 ± 7.40	21.81 ± 7.82	11.48 ± 3.63	12.02 ± 2.84
COOH-THC	0.46 ± 0.31	1.11 ± 0.68	0.84 ± 0.84	0.91 ± 0.56	0.40 ± 0.40	BQL
COOH-THC-Glu	1.91 ± 0.51	3.76 ± 1.14	1.90 ± 1.09	3.99 ± 1.90	1.17 ± 0.52	1.89 ± 1.11

For CBD, the model’s goodness-of-fit was R^2^ ≥ 0.91 at both doses. The C_max_ was 1.4 times higher at 8 mg/kg than at 4 mg/kg. The T_max_ occurred at 1 h at 4 mg/kg, and 2 h at 8 mg/kg. At 8 mg/kg, the AUC_last_ was 2.1 times, and the AUMC_last_ was 2.2 times, higher than at 4 mg/kg. At 4 mg/kg, the *t*_1/2–λz_ was 0.24 h less, and the MRT was 0.18 h less, than at 8 mg/kg; the *t*_1/2–λz_ was 0.26 h less than the MRT at 4 mg/kg and 0.20 h less than the MRT at 8 mg/kg. At 4 mg/kg, the C_max_ for one animal was at least 5.0 times higher than any other individual C_max_, and 8.0 times higher than any other animal at the 1-h timepoint. At 8 mg/kg, the C_max_ for one animal was at least 1.9 times lower, and its T_max_ was at least 6 h later, than any other individual. By the 24-h timepoint, all serum CBD concentrations were BQL and remained BQL at 4 mg/kg. On Day 7 at 8 mg/kg, all serum CBD concentrations were 2.73–4.23 ng/mL; however, on Day 14 at 8 mg/kg, the serum CBD concentration was BQL for all but one animal (less than 6.85 ng/mL).

For CBDA, the model’s goodness-of-fit was R^2^ ≥ 0.94 at both doses. The C_max_ was 1.8 times higher at 8 mg/kg than at 4 mg/kg. The T_max_ occurred at 0.5 h at 4 mg/kg, and 1 h at 8 mg/kg. At 8 mg/kg, the AUC_last_ was 3.3 times, and the AUMC_last_ was 3.9 times, higher than at 4 mg/kg. At 4 mg/kg, the *t*_1/2–λz_ was 0.26 h greater, and the MRT was 0.69 h less, than at 8 mg/kg; the *t*_1/2–λz_ was 2.91 h greater than MRT at 4 mg/kg and 1.96 h greater than MRT at 8 mg/kg. At 4 mg/kg, the C_max_ for one animal was at least 2.5 times higher than any other individual C_max_, while another was at least 3.8 times lower than any other individual C_max_. At 8 mg/kg, the individual C_max_ values ranged from 223.54 ng/mL at 1 h to 1391.74 ng/mL at 0.5 h. By the 24-h timepoint, all serum CBDA concentrations ranged from 1.92–4.73 ng/mL at 4 mg/kg and 5.47–23.34 ng/mL at 8 mg/kg. On Day 7, the serum CBDA concentration for only one individual (at 4 mg/kg) was BQL; similarly, on Day 14, the serum CBDA concentration for only one individual (at 8 mg/kg) was BQL.

For THC, the model’s goodness-of-fit was R^2^ = 0.9989 at 4 mg/kg and R^2^ = 0.8395 at 8 mg/kg. The C_max_ was 1.2 times higher at 8 mg/kg than at 4 mg/kg. The T_max_ occurred at 1 h at 4 mg/kg, and 2 h at 8 mg/kg. At 8 mg/kg, the AUC_last_ was 2.1 times, and the AUMC_last_ was 4.0 times, higher than at 4 mg/kg. At 4 mg/kg, the *t*_1/2–λz_ was 2.57 h less, and the MRT was 2.35 h less, than at 8 mg/kg; the *t*_1/2–λz_ was 0.70 h less than the MRT at 4 mg/kg and 0.48 h less than the MRT at 8 mg/kg. The C_max_ for one animal that received 4 mg/kg was BQL at all timepoints, while another that received 8 mg/kg was the only animal with detectable serum THC concentrations by the 8-h timepoint (less than 2.99 ng/mL). All serum THC concentrations were BQL for all animals at the 24-h, 7-day, and 14-day timepoints.

For THCA, the model’s goodness-of-fit was R^2^ ≥ 0.94 at both doses. The C_max_ was 1.7 times higher at 8 mg/kg than at 4 mg/kg. The T_max_ occurred at 1 h at both doses. At 8 mg/kg, the AUC_last_ was 2.4 times, and the AUMC_last_ was 2.9 times, higher than at 4 mg/kg. At 4 mg/kg, the *t*_1/2–λz_ was 0.76 h less, and the MRT was 1.11 h less, than at 8 mg/kg; the *t*_1/2–λz_ was 0.30 h less than the MRT at 4 mg/kg and 0.65 h less than the MRT at 8 mg/kg. At 4 mg/kg, the C_max_ for one animal was at least 2.5 times higher than any other individual C_max_ and 5.8 times higher than any other animal at the 0.5-h timepoint; by the 2-h timepoint, the serum THCA concentration of that animal was less than the mean. The C_max_ for another animal at 4 mg/kg was 5.3 times lower than any other C_max_. On Day 7, the serum THCA concentration was BQL for all but one animal (less than 1.50 ng/mL) at 4 mg/kg and none of the animals at 8 mg/kg; however, on Day 14, all but one individual at 4 mg/kg, and only one animal 8 mg/kg, had serum THCA concentrations less than 1.00 ng/mL.

For 7-COOH-CBD, the model’s goodness-of-fit was R^2^ ≥ 0.99 at both doses. The C_max_ was 2.9 times higher at 8 mg/kg than at 4 mg/kg. The T_max_ occurred at 1 h at 4 mg/kg and 2 h at 8 mg/kg. At 8 mg/kg, the AUC_last_ was 2.4 times, and the AUMC_last_ was 2.3 times, higher than at 4 mg/kg. At 4 mg/kg, the *t*_1/2–λz_ was 1.77 h greater, and the MRT was 0.44 h greater, than at 8 mg/kg; the *t*_1/2–λz_ was 5.25 h greater than the MRT at 4 mg/kg and 3.92 h greater than the MRT at 8 mg/kg. At 4 mg/kg, the C_max_ for one animal was at least 2.3 times higher than any other individual C_max_ and 4.6 times higher than any other animal at the 1-h timepoint. The C_max_ and T_max_ results at 8 mg/kg ranged widely by individual: 151.02 ng/mL at 2 h, 103.05 ng/mL at 4 h, 31.99 ng/mL at 2 h, and 16.70 ng/mL at 8 h. Once the serum 7-COOH-CBD concentration was above the BQL, it did not fall below BQL in any subsequent measurements.

#### 3.1.2 Serum biochemistry and complete blood count

A total of 22 biochemistry analytes were evaluated on Days 0, 1, and 14 and summarized in Supplementary Table S2. Of the liver parameters, alanine transaminase and total bilirubin did not significantly differ by time or sex. ALP (*p* = 0.0218) and gamma-glutamyl transferase (GGT; *p* = 0.0087) significantly differed by sex. The mean ALP was consistently higher in males than females. One male at 4 mg/kg (898 U/L) and another at 8 mg/kg (954 U/L) were higher than the recommended ALP reference range at Day 0, and the other male at 8 mg/kg (1,392 U/L) was higher than the recommended ALP reference range on Day 1. ALP was within the recommended reference range for all individuals on Day 14. GGT was consistently higher in all males than females, but all values were still within the recommended reference range. AST significantly differed over time (*p* = 0.0094). The mean AST was 1.9–2.5 times higher at Day 1 compared to Day 0 and Day 14; one individual at 4 mg/kg and two individuals at 8 mg/kg were higher than the recommended reference range at Day 1. AST was within the recommended reference range for all individuals by Day 14. All liver parameters are summarized in Table 4.

**Table 4:** Serum liver biochemistry analytes (mean ± SEM) after once daily oral administration of cannabidiol-/cannabidiolic acid-rich hemp oil at 4 or 8 mg/kg to juvenile cynomolgus macaques (*n* = 4 per dose) on Days 0, 1, and 14.

Parameter	Reference range	Day 0		Day 1		Day 14	
		4 mg/kg	8 mg/kg	4 mg/kg	8 mg/kg	4 mg/kg	8 mg/kg
Alkaline phosphatase^†^	46–875 U/L	686 ± 129	668 ± 146	594 ± 102	758 ± 241	525 ± 99	544 ± 129
Alanine transaminase	0–120 U/L	47 ± 9	66 ± 23	64 ± 15	74 ± 14	49 ± 6	64 ± 24
Aspartate transaminase^**^	16–88 U/L	33 ± 2	38 ± 3	80 ± 23	71 ± 16	33 ± 3	33 ± 3
Gamma-glutamyltransferase^‡^	21–184 U/L	97 ± 16	104 ± 23	88 ± 14	102 ± 21	91 ± 13	96 ± 20
Total bilirubin	0.00–2.00 mg/dL	0.09 ± 0.02	0.11 ± 0.03	0.11 ± 0.03	0.17 ± 0.07	0.10 ± 0.02	0.12 ± 0.03

Significantly changed over time ^**^(*p* ≤ 0.01); Significantly differed by sex ^†^(*p* ≤ 0.05), ^‡^(*p* ≤ 0.01). Results were rounded to match the reference range.

The complete blood count parameters are summarized in Supplementary Table S3. Three Day 14 samples (2 females at 4 mg/kg, 1 female at 8 mg/kg) submitted for hematologic analysis were reported as frozen by the laboratory, invalidating the results, and excluding them from analysis. No parameters significantly differed by dose.

## 4 Discussion

Few studies have been published regarding the PK or use of CBD in NHP. The PK of intravenous (1.4 mg/kg; *n* = 2) and oral (114 mg/kg; *n* = 1) CBD administration in rhesus macaques (*Macaca mulatta*) were reported while evaluating if electron-capture gas chromatography could be used to analyze serum CBD concentrations (77). The PK of a combination product, CBD (3 mg/kg) and THC (1 mg/kg), administered IM daily for 4 months were determined in common squirrel monkeys (*Saimiri sciureus*; *n* = 4) (78). High-dose CBD (150–300 mg/kg) administered intravenously to rhesus macaques (*n* = 12) resulted in a median lethal dose of 212 mg/kg, and side effects included tremors, emesis, and abnormal respiratory rate (66). Finally, multiple NHP studies have demonstrated that CBD can attenuate the behavioral and neurological (cognition, memory, task performance) effects of THC (78–82).

In addition to NHP, PK studies of CBD or CBDA have been conducted in dogs (28, 55, 70, 71), cats (55, 61, 83), humans (84–86), horses (87, 88), cows (89–91), rabbits (92), guinea pigs (93), rats (94), mice (51), and parrots (95). The CBD doses examined have been highly variable, ranging from 0.5–300 mg/kg in humans (96). Except for Epidiolex, an oral CBD oil, CBD and CBDA products are not FDA approved or regulated, which can affect quality and cannabinoid concentrations. Other products vary in form and administration route, including oral chews (70), oral pastes (61), oral soft gels (71), transmucosal sprays (21), transdermal gels (97), inhalational powders (98), and SC (94), IM (80), intraperitoneal (49), or intravenous (77) injections, which affect serum cannabinoid concentrations and bioavailability.

Based on the CBD:CBDA ratio of the CBD/ArHO used in this study, administration at a 4 mg/kg dose equated to approximately 2 mg/kg of each CBD and CBDA; these doses must be considered when comparing our findings with other studies, especially those that utilized pure CBD isolates. There is conflicting evidence of the pharmacokinetic interactions of CBD and CBDA when administered in a multi-cannabinoid product compared to a CBD isolate (1, 99).

### 4.1 Pharmacokinetic study

Based on our pilot study (72), low-dose human recommendations (84–86, 100), and other studies (61, 70), increased doses of 4 and 8 mg/kg/day were selected for our PK study. Throughout this study, the animals actively avoided direct-to-mouth CBD/ArHO administration and mildly hypersalivated post-administration, a reported sign of unpalatability in macaques (101). Similarly, cats were reported with signs associated with unpalatability (i.e., lip-licking, head-shaking, and drooling) post-CBD/ArHO administration (55). In addition, use of the squeeze-back cage mechanism was necessary for dosing and likely resulted in increased environmental stress compared to more passive drug delivery systems; however, no additional adverse effects were observed, supporting the relative tolerance of CBD/ArHO.

Due to the number of animals available of similar age and weight meeting inclusion criteria, sample size in this study was low; this, the need to group individuals for the PK analysis due to the frequency of cannabinoid BQL values, and drastic differences in individual C_max_ or T_max_ skewed the overall means, limited statistical power, and prevented identification and exclusion of outliers. High inter-subject variability in cannabinoid concentration has been reported previously in humans (86).

#### 4.1.1 Serum cannabinoids

While all serum cannabinoid concentrations were higher at 8 mg/kg than 4 mg/kg, serum CBDA concentration over time was the only statistically significant difference detected between doses (*p* = 0.0361). At 8 mg/kg, the C_max_ of CBD, CBDA, THC, THCA, and CBGA were less than twice those at 4 mg/kg, while the C_max_ for 7-COOH-CBD, COOH-THC, and COOH-THC-Glu were more than twice those at 4 mg/kg. While a linear relationship between dose and AUC of cannabinoids has been reported (86), other studies have reported a less than dose-proportional increase in C_max_ and AUC as dose increased, suggesting the potential of dose-based difference in bioavailability or metabolism (102). Interestingly, at twice the dose, our results found that the C_max_ for CBD and CBDA less than doubled, while the AUC was 2.1–3.3 times higher.

One PK analysis in dogs at 8 mg/kg had a C_max_ for CBD of 591 ng/mL (28), 26.5 times higher than in our subjects, indicating a higher absorption and bioavailability, or differences in metabolism, distribution, or elimination, compared to cynomolgus macaques. Cats have a lower serum concentration of cannabinoids compared to dogs but varied by study. The C_max_ of an orally administered pure CBD isolate in oil was 17.8 ng/mL at 2.5 mg/kg, 61.1 ng/mL at 5 mg/kg, and 132.6 ng/mL at 10 mg/kg (83). In another study administering CBD/ArHO at 2 mg/kg, the C_max_ of CBD was 43 ng/mL in cats, 6 times lower than dogs in the same study (55); however, this C_max_ was twice that of the C_max_ in our subjects at 8 mg/kg. In a third study administering an oral paste at approximately 2.5 mg/kg (1.37 mg/kg CBD + 1.13 mg/kg CBDA), the C_max_ of CBD was 6.6 times higher than cats receiving 2 mg/kg CBD/ArHO (55, 61). In humans, pure CBD at 1.25 mg/kg resulted in a serum CBD concentration of 37.6 ng/mL at the 2.5-h timepoint, closer to our subjects, albeit 1.7 times higher than our 8 mg/kg CBD/ArHO (86). At a 200 mg/subject dose, the C_max_ was 153 ng/mL in healthy patients (85), similar to dogs dosed with CBD/ArHO at 2 mg/kg (28, 70).

The C_max_ of CBDA in cats at 2.5 mg/kg (1.37 mg/kg CBD + 1.13 mg/kg CBDA) of oral paste was higher (1011.3 ng/mL) than for our subjects (807.33 ng/mL) at 8 mg/kg CBD/ArHO, although the AUC_last_ in our subjects was minimally higher (2759.26 ng/mL) than in cats (2638.7 ng/mL) (61). This suggests that a 4 times higher dose would be required to reach the same CBDA absorption. The C_max_ for CBDA was at more than 28.6 times that of CBD, indicating a higher oral absorption and bioavailability of CBDA, consistent with cannabis extract administration in humans (103); however, few studies have determined the therapeutic dose for CBDA. In dogs, the C_max_ for CBDA was 2–6 times higher than CBD when administered at 2 mg/kg (70, 71).

In our study, the T_max_ was 1–2 h for CBD, and 0.5–1 h for CBDA while the *t*_1/2–λz_ was 5.57–5.81 h for CBD and 6.54–6.80 h CBDA. While no animal fell BQL for CBDA by the 24-h timepoint, for CBD, two individuals at 4 mg/kg were BQL at the 8-h timepoint, and one animal at 8 mg/kg was BQL at the 12-h timepoint; thus, dosing every 6–12 h would provide better coverage. Twice daily dosing was most reported in dogs (28), cats (55), and humans (102), was therapeutically efficacious in dogs and humans, and may be a more appropriate dosing regimen for NHP.

With the exception of THCA at 4 mg/kg, the T_max_ of the cannabinoid acids (CBDA, THCA, and CBGA) were earlier than the other cannabinoids detected, indicating rapid serum absorption as also reported in mice (51); however, in our study, the t_1/2–λz_ of cannabinoid acids was longer than the corresponding neutral cannabinoid. In this study, the serum CBDA concentration was 10.9–11.2 times higher than the second highest cannabinoid, THCA, and 28.6–36.2 times higher than CBD, despite approximately equal concentrations of CBD and CBDA in the CBD/ArHO. Like CBDA, the THCA C_max_ was higher than THC, consistent with a similar study in dogs and indicating better absorption (70). As in other studies using CBD/ArHO (70, 71), serum THC concentrations were considerably lower than other metabolites, reaching less than 4.81 ng/mL even at 8 mg/kg; given the low THC concentration (0.15 weight percent) in the CBD/ArHO, this was expected.

In humans, 7-COOH-CBD is the major circulating cannabinoid (85, 86, 102). At 1.25 mg/kg of a pure CBD isolate, the serum 7-COOH-CBD (157 ng/mL) concentration was 4.2 times higher than circulating CBD (37.6 ng/mL) (86). Conversely, at 2 mg/kg CBD/ArHO, the 7-COOH-CBD C_max_ (13 ng/mL) in dogs was 9.5 times lower than that of CBD (124 ng/mL) (70), and, at 2.5 mg/kg (1.37 mg/kg CBD + 1.13 mg/kg CBDA), the 7-COOH-CBD C_max_ (41.4 ng/mL) in cats was 6.8 times lower than CBD (282 ng/mL) (61). More similarly to humans but at much lower serum concentrations, the 7-COOH-CBD C_max_ (24.19 ng/mL) in our study was 1.5 times the CBD C_max_ (15.98 ng/mL) at 4 mg/kg and 3.2 times (70.31 ng/mL) the CBD C_max_ (22.31 ng/mL CBD) at 8 mg/kg. On Days 1, 7, and 14, 7-COOH-CBD was higher than serum CBD and CBDA concentrations, potentially due to conversion to or reduced elimination of 7-COOH-CBD. Despite its persistent serum concentration, 7-COOH-CBD was not responsible for the anticonvulsant effects of CBD in animals (104).

#### 4.1.2 Serum biochemistry and complete blood count

Of the liver parameters, males had significantly higher ALP and GGT levels compared to females. Male cynomolgus macaques (after 36 months of age) and humans have also been reported to have higher ALP and GGT than females; ALP elevations also occur in young animals due to bone growth (105–107). AST was the only analyte that significantly differed over time (*p* = 0.0094). While AST is a biomarker of hepatocellular injury, elevations may also be due to normal variation, hemolysis, exercise, or muscle injury or disease. Concurrently elevated CK, as seen in 2 of the 3 animals at Day 0, often occurs in myopathies or issues with phlebotomy technique. Without elevations in alanine transaminase, high AST is less likely related to hepatopathy (108). After 2-week administration of CBD/ArHO, our results indicated that no clinically significant biochemical changes occurred over time.

Three complete blood count samples collected on Day 14 (all female, two at 4 mg/kg, one at 8 mg/kg) froze between shipment and analysis. This resulted in artificially low hematologic parameters, especially for the white blood cells. Similarly, whole-blood storage at −70°C for 15–30 days prior to a complete blood cell count significantly lowered all mean parameters other than hemoglobin and platelet count compared to fresh whole blood (109). After 2-week administration of CBD/ArHO, our overall results indicated that no clinically significant hematological changes occurred over time.

### 4.2 Other considerations

As terpenes are likely responsible for the smell and flavor of cannabinoid products, contribute to their bitter and unpleasant taste, and reduce patient compliance, the removal of terpenes from the formulation could improve palatability; however, given that terpenes may increase cannabinoid efficacy due to the entourage effect (11), this could reduce dose potency. Due to these concerns and to reduce stress associated with direct-to-mouth administration, we attempted CBD/ArHO administration in a 10-mL gelatin-based gummy vehicle; however, we encountered additional compliance issues including wide-ranging consumption times or refusal to consume the gummy at all. Alternatively, combining the CBD/ArHO with other, more palatable or aromatic substances such as peppermint oil may mask its bitter taste and improve palatability (110).

The pharmacokinetics of cannabinoids vary based on diet and feeding schedule. Feeding a high-fat diet increased serum CBD concentrations in dogs (70) and humans (102, 111). Conversely, feeding rabbits immediately after CBD/ArHO administration resulted in decreased serum CBD and CBDA concentrations (92). Unlike dogs and humans, rabbits are hindgut fermenters and consume a high-fiber diet, which may absorb the oil. Based on the results in non-hindgut fermenters, feeding our cynomolgus macaques could improve the C_max_ of cannabinoids, as in dogs and humans. On collection days, feed was provided after the 4-h collection; however, feed consumption varied among individuals over the course of each day, so some animals may have had more feed in their gastrointestinal tract than others. Providing a high-fat meal prior to dosing could decrease inter-individual variability and increase absorption.

Although cannabinoid serum accumulation did not appear to occur in our study, the once daily dosing may have been insufficient for this effect. Given that CBD was BQL by the 24-h timepoint, increased CBD/ArHO dosing frequency and duration would be necessary to demonstrate any cumulative effects on serum cannabinoid concentration. Human studies suggest a minimal to moderate CBD accumulation over time (102, 112), but metabolites such as 7-COOH-CBD have greater accumulation (86).

Therapeutic doses and serum concentrations of cannabinoids have not been determined in most species, including macaques. For CBD, the recommended therapeutic doses and serum concentrations vary in reports in other species depending on its intended use. In humans, plasma CBD concentrations of 100 ng/mL were effective in reducing seizures and doses up to 50 mg/kg had a linear increase in plasma concentration and efficacy (100). In dogs, 2 mg/kg of CBD resulted in a median C_max_ of 102 ng/mL and twice daily dosing effectively reduced osteoarthritis-related discomfort (28). In our subjects at 8 mg/kg, the C_max_ of CBD only reached 22 ng/mL, 4.5 times less, suggesting that it would be a subtherapeutic dose if efficacy is similar to dogs and humans.

A biphasic or inverted U-shaped curve effect of CBD and other cannabinoids has been reported in multiple species. While anxiolytic or anti-emetic at certain doses, at some point increasing or decreasing the dose had no effect or even exacerbated the condition (22, 113). Doses of 0.1 and 0.5 mg/kg CBDA were effective in reducing vomiting in house shrews; however, vomiting was comparable to the control at 5 mg/kg (47). Similarly, 0.01 mg/kg of oral CBDA sufficiently reduced hyperalgesia in rats but 1 mg/kg did not (49). Little additional information is currently available regarding this effect; therefore, developing the therapeutic dose of CBD and CBDA in macaques should account for these potential issues.

As they are lipid soluble, cannabinoid concentrations should also be quantified in excreta, adipose, and target tissues (114). Cannabinoids concentrations in target tissues, such as the brain (51), joints (93), or gastrointestinal tract could affect efficacy. Histopathology could help evaluate potential beneficial or deleterious effects to healthy or disease-affected tissues after CBD/ArHO administration and determine therapeutic doses (115).

Other studies have shown anatomical and physiological side effects (66), and biochemical changes (28), after 30 days of dosing. Although our study did not detect any clinically significant changes in liver parameters, changes could be seen after long-term administration; therefore, it should be used cautiously in animals with hepatopathies, prone to liver failure, or receiving other treatments that involve cytochrome P450 mechanisms. Additionally, its use in NHP breeding colonies at lower doses should be evaluated, as high doses were shown to reduce spermatogenesis (66).

### 4.3 Conclusion

Due to poor regulation of commercially available products, which are frequently mislabeled and often widely variable in cannabinoid composition, products which provide a guaranteed analysis by an independent laboratory are most reliable. Clinical studies are needed to determine the therapeutic dose of CBD and CBDA for macaques, which may differ based on the disorder targeted. Additionally, CBD/ArHO should be evaluated as an adjunctive therapy in non-human primates. Given the low serum CBD concentrations, the doses and frequency used in this study may be insufficient for a therapeutic effect; however, if CBDA has similar therapeutic benefit to CBD, then CBD/ArHO has promise. During a clinical pilot study, we observed that accessing animals for multiple daily dosing may have increased environmental stress, which may limit the usefulness of CBD/ArHO unless an alternative, palatable vehicle is developed. In addition, once daily dosing would be more convenient and increase compliance of use as some facilities do not have 24-h personnel able to medicate the animals every 6–12 h; however, our PK data suggested that once daily dosing was insufficient in maintaining serum CBD concentrations. Given the considerable inter-subject variability and differences of our results compared to other species, CBD/ArHO should be evaluated for reproducibility in other cynomolgus macaques and other NHP to determine if results are similar to our findings.

## Data availability statement

The raw data supporting the conclusions of this article will be made available by the authors, without undue reservation.

## Ethics statement

The animal study was approved by the Mannheimer Foundation’s Institutional Animal Care and Use Committee. The study was conducted in accordance with the local legislation and institutional requirements.

## Author contributions

TJ: Conceptualization, Data curation, Formal analysis, Investigation, Methodology, Project administration, Supervision, Writing – original draft, Writing – review & editing, Funding acquisition, Resources. JW: Conceptualization, Funding acquisition, Resources, Writing – review & editing. AL: Data curation, Formal analysis, Methodology, Validation, Writing – original draft. AZ: Data curation, Formal analysis, Methodology, Validation, Writing – review & editing. WB: Conceptualization, Data curation, Formal analysis, Funding acquisition, Investigation, Methodology, Project administration, Resources, Supervision, Visualization, Writing – original draft, Writing – review & editing.

## Footnotes

^1^ http://www.random.org/sequences

## Glossary

7-COOH-CBD: 7-Nor-7-carboxycannabidiol
ALP: Alkaline phosphatase
AUC_last_: Area under the curve until the last measurement
AUMC_last_: Area under the moment curve until the last measurement
AST: Aspartate aminotransferase
BQL: Below the quantification level
CBD: Cannabidiol
CBDA: Cannabidiolic acid
CBD/ArHO: Cannabidiol-/cannabidiolic acid-rich hemp oil
CBG: Cannabigerol
CBGA: Cannabigerolic acid
CBN: Cannabinol
C_max_: Maximum serum concentration
COOH-THC: 11-nor-9-Carboxy-Δ^9^-tetrahydrocannabinol
COOH-THC-Glu: 11-Nor-9-carboxy-Δ^9^-tetrahydrocannabinol-Glucuronide
GGT: Gamma-glutamyl transferase
IM: Intramuscularly
MRT_last_: Mean residence time until the last measurement
NHP: Nonhuman primate
PK: Pharmacokinetic
R^2^: Coefficient of determination
SC: Subcutaneously
*t*_1/2–λz_: Half-life of the terminal phase
THC: ∆^9^-tetrahydrocannabinol;
THCA: ∆^9^-tetrahydrocannabinolic acid
T_max_: Time to maximal serum concentration
