# Supplementary material for: Pharmacokinetics of cannabidiol-/cannabidiolic acid-rich hemp oil in juvenile cynomolgus macaques (Macaca fascicularis)
Source: Front Vet Sci. 2023 Nov 29;10:1286158. doi: 10.3389/fvets.2023.1286158 (PMC10716325; doi:10.3389/fvets.2023.1286158)
Supplement: Supplementary file 1 [file Data_Sheet_1.pdf]

Supplementary Material

Pharmacokinetics of cannabidiol-/cannabidiolic acid-rich hemp oil in juvenile cynomolgus macaques (*Macaca fascicularis*)

Tinika N Johns*, Joseph J Wakshlag, Alexander V Lyubimov, Alexander Zakharov, Wesley M Burnside

***Correspondence:** Corresponding Author: tjohns@hamanranch.org

Supplemental Table S1 | Guaranteed oil analysis of the cannabidiol-/cannabidiolic acid-rich hemp oil used in this study performed by ProVerde Laboratories using liquid chromatography (plant-based cannabinoids) or head-space gas chromatography (terpenes). The data were compared to certified reference standards at known concentrations. All terpene values are semiquantitative estimates based on recorded peak areas relative to calibration data. The limit of quantitation (LOQ) was equal to 0.01 weight percent. Samples were considered none detected (ND) if less than half of the LOQ.

Cannabinoid	Concentration (mg/mL [weight %])	Terpenes	Weight (%)
Cannabidiol (CBD)	28.53 (3.09)	Myrcene	0.15
Cannabidiolic acid (CBDA)	34.01 (3.69)	Isopulegol	0.00
Δ^9-tetrahydrocannabinol (THC)	1.34 (0.15)	Linalool	0.02
Δ^9-Tetrahydrocannabinolic acid (THCA)	1.26 (0.14)	B-caryophyllene	0.03
Cannabigerolic acid (CBGA)	0.86 (0.09)	Humulene	0.01
Cannabichromene (CBC)	1.11 (0.12)	Camphene	0.00
Cannabinol (CBN)	< LOQ (< 0.01)	B-pinene	0.03
Cannabigerol (CBG)	0.36 (0.04)	Eucalyptol	0.01
Cannabidivarin (CBDV)	< LOQ (< 0.01)	A-pinene	0.10
Δ^9-tetrahydrocannabivarin (THCV)	ND	Limonene	0.02
Δ^8-tetrahydrocannabinol (D8-THC)	ND		
Exo-tetrahydrocannabinol (exo-THC)	ND		

Supplemental Table S2 | Serum biochemistry analytes (mean ± SEM) after once daily oral administration of cannabidiol-/cannabidiolic acid-rich hemp oil at 4 or 8 mg/kg to juvenile cynomolgus macaques (*n* = 4 per dose) on Days 0, 1, and 14. Analytes were grouped by organ (liver, kidney, pancreas), electrolytes and minerals, proteins, or other. Results were rounded to match the reference range.

Parameter	Reference Range	Day 0		Day 1		Day 14	
		4 mg/kg	8 mg/kg	4 mg/kg	8 mg/kg	4 mg/kg	8 mg/kg
**Liver**							
Alkaline phosphatase†	46–875 U/L	686 ± 129	668 ± 146	594 ± 102	758 ± 241	525 ± 99	544 ± 129
Alanine transaminase	0–120 U/L	47 ± 9	66 ± 23	64 ± 15	74 ± 14	49 ± 6	64 ± 24
Aspartate transaminase**	16–88 U/L	33 ± 2	38 ± 3	80 ± 23	71 ± 16	33 ± 3	33 ± 3
Gamma-glutamyltransferase‡	21–184 U/L	97 ± 16	104 ± 23	88 ± 14	102 ± 21	91 ± 13	96 ± 20
Total bilirubin	0.00–2.00 mg/dL	0.09 ± 0.02	0.11 ± 0.03	0.11 ± 0.03	0.17 ± 0.07	0.10 ± 0.02	0.12 ± 0.03
**Kidney**							
Blood urea nitrogen*	5–31 mg/dL	21 ± 2	21 ± 1	18 ± 2	17 ± 1	20 ± 3	21 ± 1
Creatinine	0.4–1.6 mg/dL	0.80 ± 0.00	0.85 ± 0.03	0.78 ± 0.03	0.80 ± 0.04	0.85 ± 0.03	0.80 ± 0.00
**Pancreas**							
Amylase	50.4–660.0 U/L	360.3 ± 41.6	317.8 ± 18.4	376.0 ± 74.2	297.5 ± 17.7	388.5 ± 91.1	317.8 ± 26.0
Lipase*	35–256 U/L	22 ± 8	20 ± 2	13 ± 4	14 ± 1	26 ± 13	22 ± 3
**Electrolytes & Minerals**							
Calcium*	8–12 mg/dL	10 ± 0	11 ± 0	9 ± 0	10 ± 0	10 ± 0	10 ± 0
Chloride**	84–126 mmol/L	104 ± 1	105 ± 1	108 ± 0	107 ± 1	105 ± 1	106 ± 1
Potassium	2.3–6.7 mmol/L	5.1 ± 0.1	5.1 ± 0.2	4.9 ± 0.1	5.2 ± 0.2	4.8 ± 0.2	4.6 ± 0.1
Magnesium	0.04–1.84 mmol/L	0.73 ± 0.03	0.76 ± 0.04	0.71 ± 0.03	0.71 ± 0.01	0.71 ± 0.05	0.72 ± 0.02
Sodium	102.0–166.0 mmol/L	146.5 ± 1.0	147.9 ± 0.8	147.3 ± 0.6	148.7 ± 0.5	148.1 ± 2.6	146.0 ± 0.7
Phosphorus	1.4–7.5 mg/dL	4.9 ± 0.7	4.9 ± 0.3	4.2 ± 0.2	4.2 ± 0.2	5.2 ± 0.5	5.2 ± 0.4
**Proteins**							
Albumin	2.63–8.63 g/dL	3.25 ± 0.10	3.45 ± 0.13	3.13 ± 0.12	3.30 ± 0.14	3.08 ± 0.17	3.40 ± 0.07
Globulins**‡	1.7–4.5 g/dL	4.4 ± 0.2	4.5 ± 0.2	4.0 ± 0.2	4.1 ± 0.2	4.1 ± 0.3	4.2 ± 0.1
Total protein**	5.90–9.61 g/dL	7.65 ± 0.09	7.93 ± 0.18	7.15 ± 0.19	7.35 ± 0.27	7.18 ± 0.31	7.60 ± 0.08
**Other**							
Cholesterol**	38–222 mg/dL	111 ± 11	113 ± 7	92 ± 8	94 ± 10	109 ± 11	118. ± 8
Creatine kinase*	0–3003 U/L	271 ± 105	205 ± 88	497 ± 163	545 ± 218	224 ± 44	129 ± 13
Glucose	37.00–131.33 mg/dL	96.75 ± 11.05	90.25 ± 8.27	94.00 ± 17.95	89.00 ± 14.53	110.50 ± 17.63	98.25 ± 8.06
Triglycerides*	6–123 mg/dL	79 ± 15	58 ± 6	59 ± 13	46 ± 9	90 ± 13	59 ± 2

Significantly changed over time *($p \leq 0.05$), **($p \leq 0.01$); Significantly differed by sex †($p \leq 0.05$), ‡($p \leq 0.01$)

Supplementary Table S3 | Complete blood count parameters (mean ± SEM) after once daily oral administration of cannabidiol-/cannabidiolic acid-rich hemp oil at 4 or 8 mg/kg to juvenile cynomolgus macaques (n = 4 per dose) on Days 0, 1, and 14. Parameters were grouped by were grouped by white blood cells, red blood cells, and platelets. Results were rounded to match the reference range.

**Parameter**	**Reference Range**	**Day 0**		**Day 1**		**Day 14**	
		**4 mg/kg**	**8 mg/kg**	**4 mg/kg**	**8 mg/kg**	**4 mg/kg**	**8 mg/kg**
White blood cells	3.50–21.10 K/µL	11.47 ± 1.86	10.69 ± 1.16	14.87 ± 3.27	12.10 ± 2.67	7.82 ± 0.63	11.20 ± 1.87
Neutrophils	0.20–14.00 K/µL	4.63 ± 1.02	4.26 ± 1.39	8.31 ± 3.15	6.83 ± 1.92	3.13 ± 0.10	5.35 ± 1.12
Lymphocytes	1.00–17.40 K/µL	5.20 ± 0.63	5.16 ± 0.40	4.80 ± 0.33	4.29 ± 0.93	1.96 ± 1.11	3.34 ± 1.23
Monocytes	0.00–1.30 K/µL	1.00 ± 0.35	0.77 ± 0.09	1.26 ± 0.25	0.55 ± 0.09	0.52 ± 0.07	0.74 ± 0.07
Eosinophils	0.00–2.00 K/µL	0.36 ± 0.10	0.29 ± 0.11	0.22 ± 0.07	0.25 ± 0.09	0.21 ± 0.14	0.37 ± 0.08
Basophils	0.00–2.96 K/µL	0.29 ± 0.06	0.24 ± 0.05	0.26 ± 0.06	0.20 ± 0.03	0.14 ± 0.00	0.30 ± 0.05
Red blood cells**	3.50–7.80 M/µL	6.74 ± 0.24	6.47 ± 0.07	6.05 ± 0.14	6.15 ± 0.11	6.46 ± 0.12	6.15 ± 0.08
Hemoglobin**	8.2–15.9 g/dL	13.9 ± 0.3	13.1 ± 0.4	12.6 ± 0.3	12.4 ± 0.5	12.8 ± 0.4	12.6 ± 0.4
Hematocrit**	24.0–49.6 %	51.8 ± 1.6	48.9 ± 1.6	46.2 ± 0.9	46.3 ± 1.7	49.5 ± 2.5	47.6 ± 0.8
Mean cell volume	46.0–98.5 fL	77.0 ± 0.6	75.5 ± 2.4	76.5 ± 0.7	75.3 ± 2.4	76.6 ± 2.4	77.4 ± 0.4
Mean cell height	14.9–27.7 pg	20.7 ± 0.3	20.2 ± 0.7	20.9 ± 0.3	20.2 ± 0.7	20.6 ± 0.2	20.9 ± 0.3
Mean cell hemoglobin concentration	25.8–36.4 g/dL	26.9 ± 0.2	26.7 ± 0.2	27.3 ± 0.2	26.8 ± 0.3	27.0 ± 0.7	27.1 ± 0.3
Red blood cell distribution width**	10.9–27.0 %	17.4 ± 0.5	18.3 ± 0.8	17.4 ± 0.8	18.1 ± 0.7	19.2 ± 1.5	18.7 ± 0.6
Platelets†	195–734 K/µL	334 ± 46	345 ± 51	280 ± 30	284 ± 45	386 ± 42	374 ± 37
Mean platelet volume	5.0–20.0 fL	13.1 ± 1.2	12.0 ± 1.1	12.5 ± 0.9	12.6 ± 1.3	11.8 ± 1.2	10.9 ± 0.4

Significantly changed over time **($p \leq 0.01$); Significantly differed by sex †($p \leq 0.05$)
